# Immune Modulating Effects of NKT Cells in a Physiologically Low Dose *Leishmania major* Infection Model after αGalCer Analog PBS57 Stimulation

**DOI:** 10.1371/journal.pntd.0002917

**Published:** 2014-06-26

**Authors:** Klaus G. Griewank, Beate Lorenz, Michael R. Fischer, Louis Boon, Susanna Lopez Kostka, Esther von Stebut

**Affiliations:** 1 Department of Dermatology, University Medical Center, Johannes Gutenberg-University, Mainz, Germany; 2 Department of Dermatology, University Duisburg-Essen, University Hospital Essen, Essen, Germany; 3 Bioceros, Utrecht, The Netherlands; Institut Pasteur, France

## Abstract

Leishmaniasis is a parasitic infection affecting ∼12 million people worldwide, mostly in developing countries. Treatment options are limited and no effective vaccines exist to date. Natural Killer T (NKT) cells are a conserved innate-like lymphocyte population with immunomodulating effects in various settings. A number of reports state a role of NKT cells in different models of *Leishmania* infection. Here, we investigated the effect of NKT cells in a physiologically relevant, intradermal low dose infection model. After inoculation of 10^3^ infectious-stage *L. major*, comparable numbers of skin-immigrating NKT cells in both susceptible BALB/c mice and resistant C57BL/6 mice were noted. Compared to their wild type counterparts, NKT cell-deficient mice on a C57BL/6 background were better able to contain infection with *L. major* and showed decreased IL-4 production in cytokine analysis performed 5 and 8 weeks after infection. Low doses of the NKT cell stimulating αGalCer analog PBS57 applied at the time of infection led to disease exacerbation in C57BL/6 wild-type, but not NKT-deficient mice. The effect was dependent both on the timing and amount of PBS57 administered. The effect of NKT cell stimulation by PBS57 proved to be IL-4 dependent, as it was neutralized in IL-4-deficient C57BL/6 or anti-IL-4 antibody-treated wild-type mice. In contrast to C57BL/6 mice, administration of PBS57 in susceptible BALB/c mice resulted in an improved course of disease. Our results reveal a strain- and cytokine-dependent regulatory role of NKT cells in the development of immunity to low dose *L. major* infections. These effects, probably masked in previous studies using higher parasite inocula, should be considered in future therapy and immunization approaches.

## Author Summary

Cutaneous leishmaniasis is a disease affecting about 12 million people worldwide. It is transmitted by a sand fly and primarily affects people in developing countries. To date there are no effective vaccines. Many of the treatments available have serious side effects and resistance mechanisms are becoming an increasingly prevalent problem. Natural killer T (NKT) cells are a unique T cell population recognizing glycolipids. Their role in immune processes, especially in infectious diseases, is incompletely understood. In the current study, we investigated the role of NKT cells in *Leishmania* infections in detail. We found that NKT cells can significantly alter the development of immunity, however in different directions depending on the host's genetic background. Their natural effect on infection can be increased when applying the stimulating antigen alpha-Galactosyl-Ceramide (αGalCer) or its analogs (in our study PBS57). Our results show that the effect of these cells in resistant mice (which are generally reminiscent of the situation in humans) is largely mediated by cytokine secretion, in particular IL-4, a Th2 cytokine. We conclude that NKT cells influence the course of *Leishmania* infection and that therapeutically modulating their function could be beneficial both to treat existing infections, as well as potentially develop desperately needed, effective vaccines.

## Introduction

Leishmaniasis is a parasitic disease which is caused by a variety of *Leishmania spp.* and affects about 12 million people worldwide. There are approximately 2 million people newly infected every year. Transmitted by a sand fly, it primarily affects people in (sub-) tropical climates. Depending on the genetic background and the immune status of the patient, the disease can have various clinical presentations. It can be primarily cutaneous, mucocutaneous, multilocular, chronic, or recurrent and in severe cases develop visceral forms. About 90% of infected individuals suffer from cutaneous leishmaniasis (CL), which often heals with disfiguring scars [1]. Primarily affecting poorer populations, there is little incentive for drug and vaccine development. Leishmaniasis is considered a neglected disease by the WHO.

Experimentally, leishmaniasis has long been used as an immunologic model. Infected mouse strains can either develop a Th1/Tc1-driven immune response successfully containing infection or a Th2/Th17/Treg response, ultimately succumbing to uncontrolled parasite loads. A better understanding of the disease and the immunologic mechanisms involved will hopefully result in improved treatment options and an effective vaccine.

Natural killer T (NKT) cells are a subset of T cells first identified in the early 90s^2^. Obtaining their name because they express a subset of receptors primarily expressed on natural killer (NK) cells, they also have a number of other unique properties which distinguish them from conventional T cells. They harbor a restricted set of T cell receptors and recognize endogenous and exogenous lipid antigens presented by a MHC class 1b molecule named CD1d [2], [3]. They also have an effector phenotype and function, readily able to secrete considerable amounts of both IL-4 and IFNγ upon stimulation [4]. They have been shown to regulate and influence the immune response to a variety of infectious agents being activated either by direct recognition of bacterial lipid antigens or in a CD40-, IL-12-dependent manner by dendritic cells (DC) presenting endogenous lipids [5]. The strongest known ligand activating NKT cells is αGalactoysl-Ceramide (αGalCer) [6]. Stimulation of NKT cells by αGalCer can lead to considerable cytokine secretion when applied in small quantities and has been used as a modulator of immune responses in a variety of antitumor, autoimmune and infectious experimental models [7]–[9].

A number of studies have addressed the role of NKT cells in the immune response to *Leishmania* [10]–[17]. In visceral leishmaniasis, NKT cells were shown to recognize a *L. donovani* lipophosphoglycan and an impaired immune response in NKT deficient CD1d^−/−^ BALB/c mice was noted [18]. *L. donovani* infection causing IFNγ secretion by NKT cells through liver SIRP (signal regulatory protein α) upregulation was also reported [10]. In cutaneous leishmaniasis, infections with >10^6^ *L. major* parasites (s.c. or i.v.) showed delayed parasite clearance in NKT cell-deficient CD1d^−/−^ and Jα18^−/−^ mice [13]. In summary, the results of these studies are variable and do not provide a coherent understanding of the role of NKT cells in leishmaniasis. All *in vivo* studies reported, with the exception of vaccine trials by Dondji et al. [19], used supraphysiological high dose parasite inocula, applying >10^6^ promastigotes at the time of infection.

In our study we analyzed the effect of NKT cells in a physiologically relevant model of cutaneous leishmaniasis applying 10^3^ infectious stage *L. major* promastigotes intradermally into the ear. Our results differ considerably from prior high dose inocula studies and show effects that point toward an important influence of NKT cells on the development of protective immunity against *Leishmania*. This clear role of NKT cells for parasite clearance could be important for the development of new treatment options or an effective vaccine.

## Materials and Methods

### Mice

C57BL/6, Jα18^−/−^, CD1d^−/−^, and IL-4^−/−^ mice (all C57BL/6 background), and BALB/c mice were housed under specific pathogen-free conditions in the animal care facility in Mainz.

### Ethics statement

All animal experiments were conducted in accordance with federal guidelines and approved by the ethical committee of the state of Rheinland Pfalz (according to §8 Abs. 1 des Tierschutzgesetzes, Landesuntersuchungsamt, approval #LUA 23 170–07/G07-1-022).

### Parasites and infection

Metacyclic promastigotes of *L. major* clone VI (MHOM/IL/80/Friedlin) were prepared as described previously [20]. Groups of three to five mice were infected with low-dose (10^3^) inocula in a volume of 20 µl by intradermal injection into ear skin applying 0.3 mm diameter needles. Lesion volumes were measured weekly in three dimensions and are reported as ellipsoids: [(a/2×b/2×c/2)×4/3π]. Parasite burdens were enumerated using a limiting dilution assay [20]. All experiments used an artificial variant of αGalCer termed PBS57 [21]. It was applied i.p. after dilution in 200 µl PBS at the time of treatment or as referred to in the manuscript. Control groups received 200 µl PBS i.p. IL-4 neutralizing antibodies (clone 11B11) were applied i.p. at 1 mg/200 µl, one day before and one day after infection.

### Cytokine secretion profiles

To measure antigen-specific cytokine production, retroauricular LNs were isolated and single cell suspensions prepared. One million LN cells in 200 µl complete RPMI 1640 medium (BioWhittaker) were cultured in the presence of 25 µg/ml SLA. Supernatants were harvested 48 h after stimulation and analyzed with ELISAs specific for IFNγ (R&D Systems), IL-4 and IL-10 (BD). IFNγ and IL-4 levels in serum samples were measured the same way.

### Single cell preparation

The livers were harvested, minced, and the tissue pieces strained through 70-µm mesh and pelleted by centrifugation. The cell pellet was resuspended and lymphocytes isolated by Percoll gradient, spun at 200 g. Spleen and lymph node cells were isolated by mechanically grinding the tissue through 70-µm cell strainers.

### Cell isolation from infected mouse ears

Ears infected with *L. major* were excised, soaked in 70% ethanol, and washed with PBS. The ears were split into halves and placed in 0.5 mg/ml liberase (Sigma-Aldrich) diluted in RPMI 1640 with 5% penicillin/streptomycin for 1.5 h at 37°C. Liberase was inactivated by adding complete RPMI 1640 containing 5% FCS. The ears were put into 50 µM Medicon homogenizers (BD) with 1 ml of complete RPMI 1640 and homogenized in a Medimachine (BD) for 7 min. This was then passed through a 70 µm-pore size filter and centrifuged at 200 g for 8 min. Cells were resuspended in PBS and labeled with antibodies for flow cytometry.

### Antibodies and flow cytometry

The following antibodies were purchased from BD: PerCP-Cy5.5 rat anti–mouse CD8α (53–6.7), PE rat anti–mouse CD4 (L3T4). NKT cells were stained with APC-labeled, αGalCer analog PBS57-loaded CD1d tetramers as previously described [22]. Flow cytometry analysis was performed using a FACSCalibur or LSRII cytometer (BD) and FlowJo software (Tree Star). Cells were gated via FSC/SSC for viable cells, concentrating on lymphocytes.

### Statistical analysis

Statistical analysis was performed using StatView software and Students t-test.

## Results

### Monitoring NKT cell numbers in different tissues during the course of infection after intradermal *L. major* inoculation of C57BL/6 and BALB/c mice

**Figure 1:**
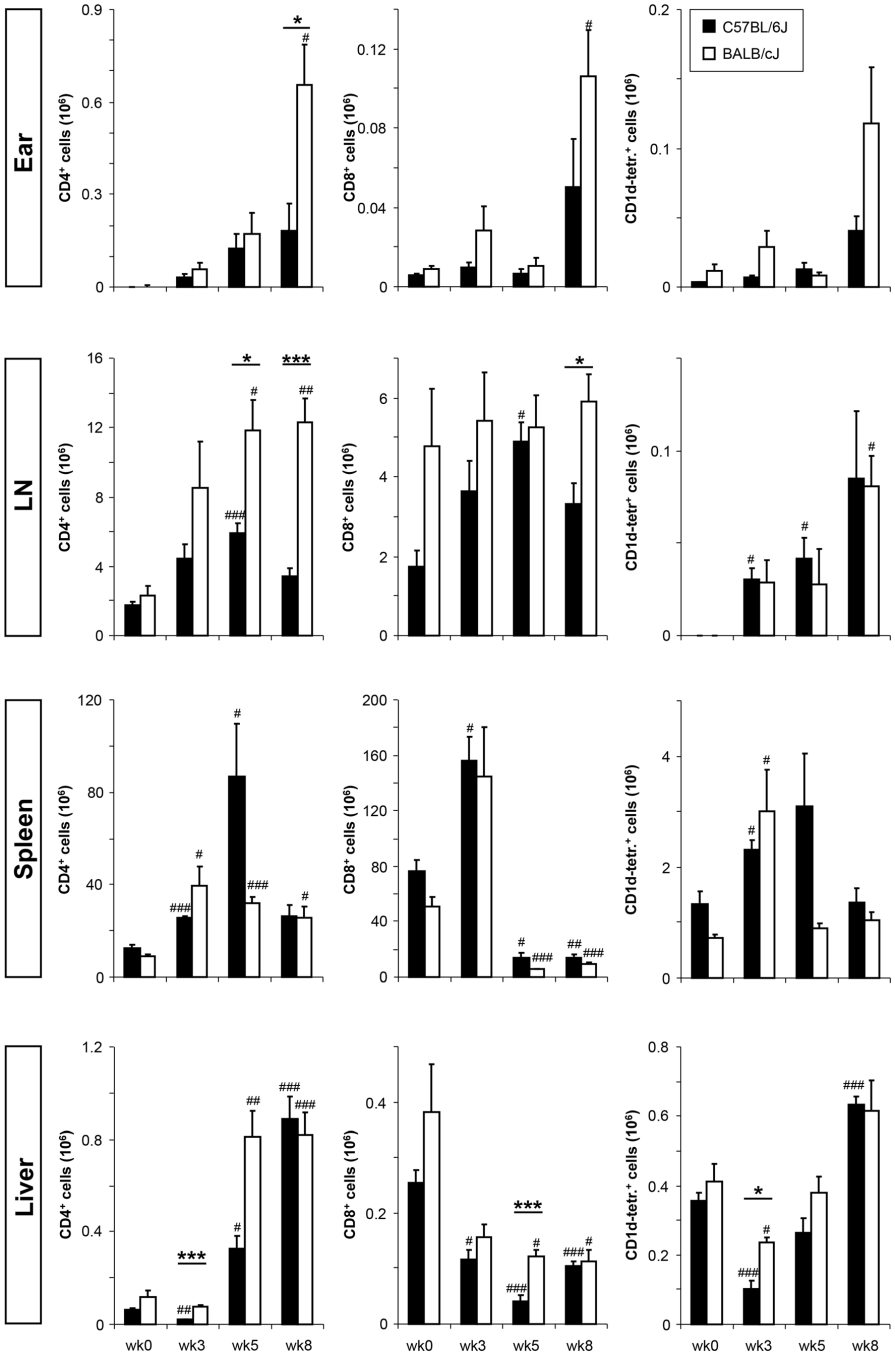
T and NKT cell numbers during the course of infection. The numbers of CD4, CD8, and NKT cells were analyzed during the course of low dose *L. major* infection in C57BL/6 and BALB/c mice. Cell frequencies were assessed by flow cytometry and numbers calculated for the ear, lymph node (LN), spleen, and liver in weeks 0, 3, 5, and 8. Significant differences between C57BL/6 and BALB/c mice at different time points are indicated by lines above bar graphs with *, **, and *** referring to p-values of ≤0.05, ≤0.005, and ≤0.002, respectively. Statistically significant changes in cell number compared to week 0 are shown as #, further statistical data is presented in Table S1 (n = 2 with 3–8 mice per group).

Post intradermal inoculation of 1,000 *L. major* in the ear pinna of C57BL/6 and BALB/c mice, we assessed the number of NKT cells at different time points in the lymph nodes, spleen and liver (Figure 1, additional statistical information in Table S1). As expected, at later time points of infection, significant increases in the amount of CD4 and CD8 T cells in the lymph nodes and ears of C57BL/6 and BALB/c groups were noted. NKT cell numbers were measured applying CD1d-PBS57 tetramers. Interestingly, in parallel to conventional T cells, NKT numbers were also increased at later time points in infected skin sites, the draining lymph nodes and the liver. Strain-dependent differences were not apparent.

### Decreased severity and duration of disease in *L. major*-infected NKT cell-deficient Jα18^−/−^ and CD1d^−/−^ C57BL/6 mice

**Figure 2:**
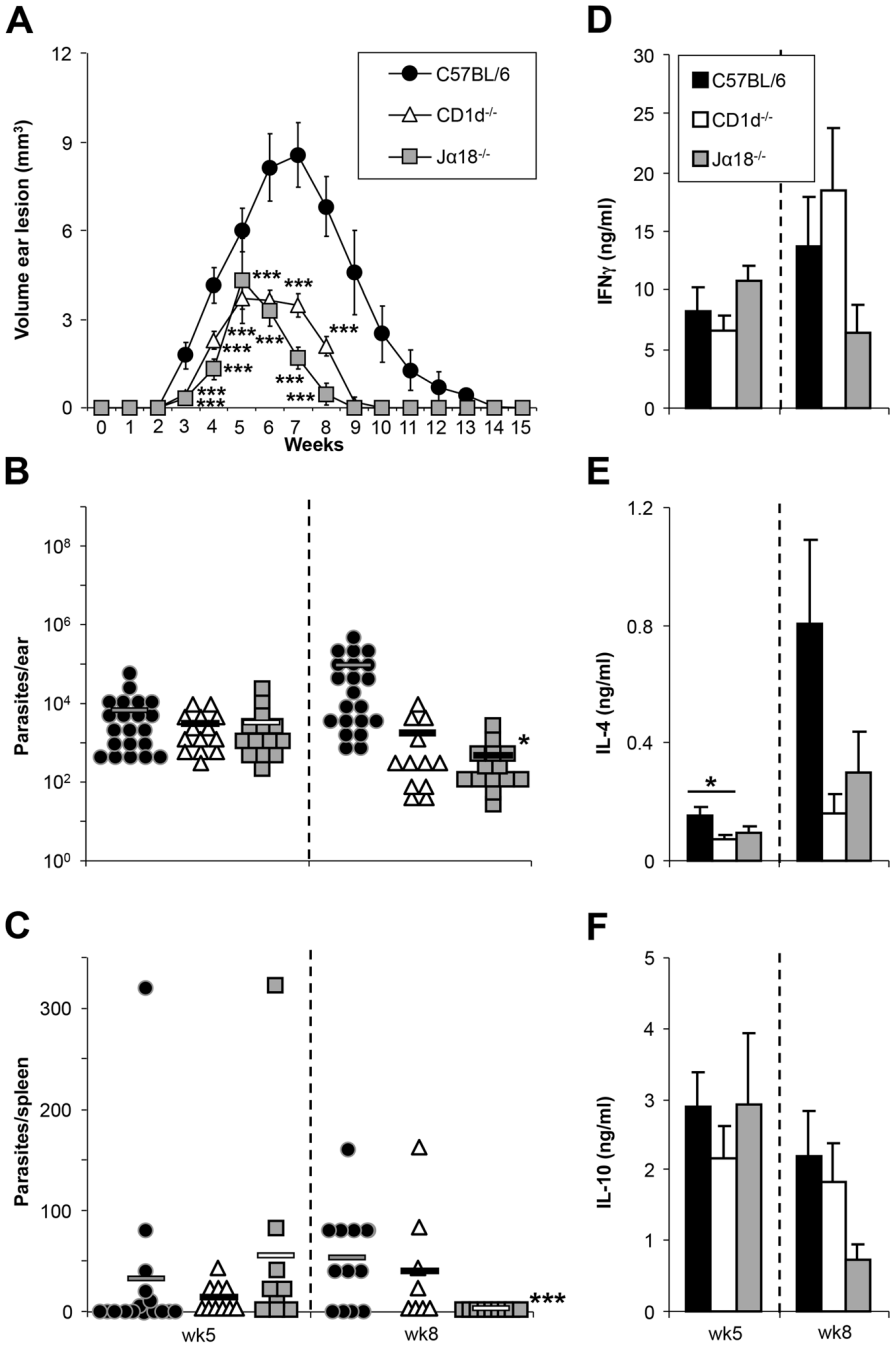
Course of infection in NKT cell-deficient mice. **A.** Ear lesion volumes of wild type C57BL/6 and NKT cell deficient CD1d^−/−^ and Jα18*^−/−^* mice infected with physiological low dose inocula (10^3^ metacyclic *L. major* promastigotes). **B+C,** Parasite loads in the ear and spleen were measured at 5 and 8 weeks post infection. **D–F,** Levels of IFNγ (D), IL-4 (E) and IL-10 (F) were assessed after restimulation with soluble *Leishmania* antigen (SLA). Significant differences to C57BL/6 wild type mice are labeled with *, **, and *** referring to p-values of ≤0.05, ≤0.005, and ≤0.002, respectively (n≥3 independent experiments with ≥3 animals per group (A, B, C) and ≥9 animals per group (D, E, F).

The course of infection in Jα18^−/−^ and CD1d^−/−^ compared to C57BL/6 wild-type mice was analyzed post intradermal injection of 1,000 *L. major* in the ear pinna. Both Jα18^−/−^ and CD1d^−/−^ mice lack the majority of NKT cells, with slight differences: Jα18^−/−^ lack those containing the invariant Vα14-Jα18 T cell receptor (TCR) alpha chain, CD1d^−/−^ lack all T cells selected by and recognizing CD1d. Both mouse strains showed a less severe course of infection than wild-type C57BL/6 controls as assessed by significantly decreased lesion volumes at different time points (Figure 2A). Parasite burdens assessed in the ear and spleen showed a similar trend with lower parasite levels in NKT cell-deficient mice. Differences between Jα18^−/−^ and C57BL/6 groups reached statistical significance (Figure 2B and 2C). Antigen-specific cytokine production was assessed after restimulation of draining LN cells (Figure 2D, 2E, and 2F respectively). A consistent decrease of IL-4 secretion in both Jα18^−/−^ and CD1d^−/−^ LN cells compared with C57BL/6 cells was noted in both weeks 5 and 8.

### Intraperitoneal αGalCer analog PBS57 administration leads to disease exacerbation in *L. major*-infected C57BL/6 mice

**Figure 3:**
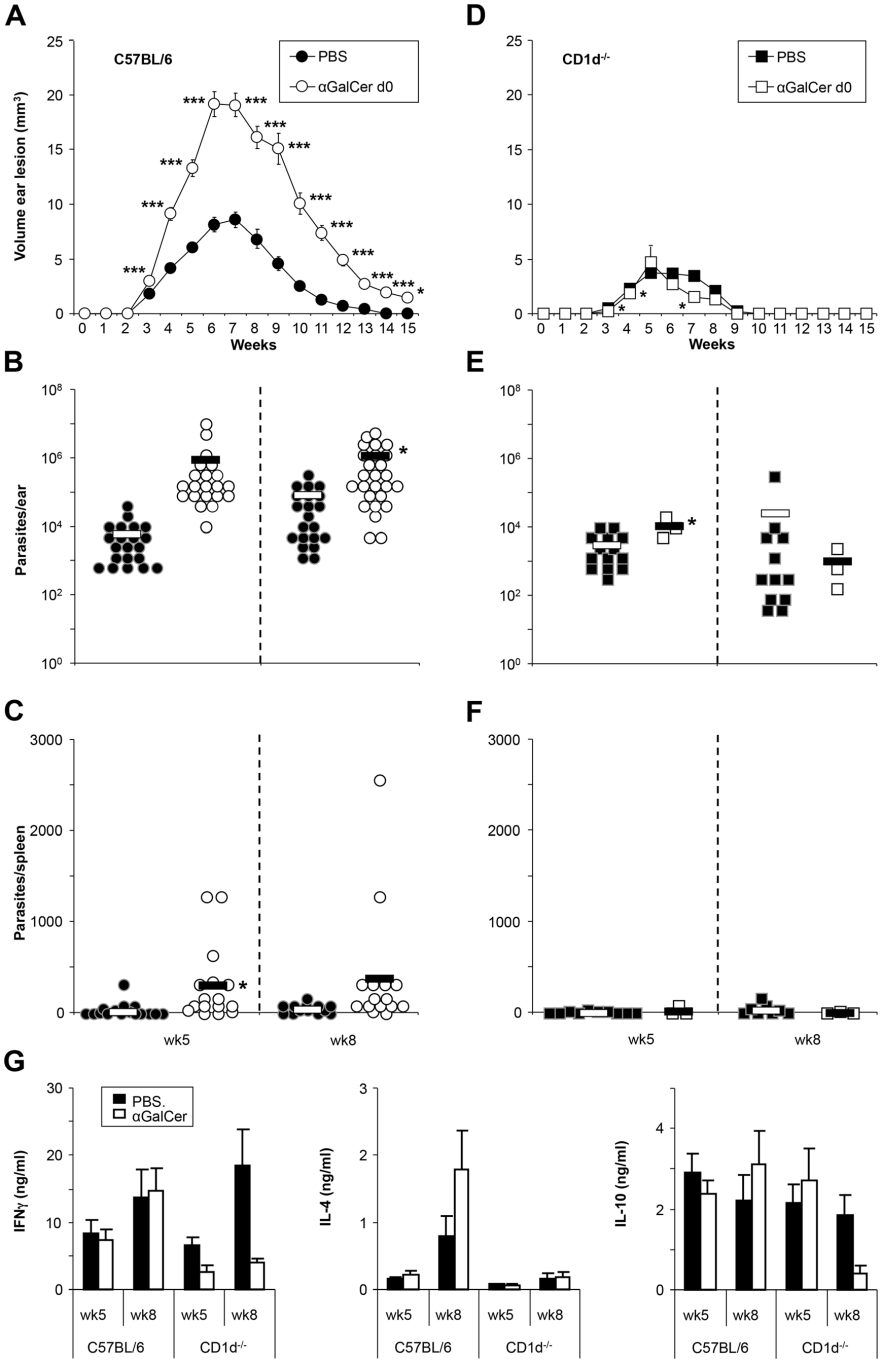
αGalCer analog PBS57 application alters the course of infection. **A,D.** Ear lesion volumes of wild type C57BL/6 (A) and NKT cell-deficient CD1d^−/−^ (D) mice infected with low dose *L. major* promastigotes with or without additionally receiving 100 ng αGalCer at the time of infection. **B, C, E, F.** Parasite loads in the ear (B,E) and spleen (C, F) measured at 5 and 8 weeks post infection in C57BL/6 (B, C) or CD1d^−/−^ (E, F) mice treated with or without αGalCer analog PBS57. G. Levels of IFNγ, IL-4, and IL-10 measured after restimulation with SLA. Significant differences to C57BL/6 control mice are labeled with *, **, and *** referring to p-values≤0.05, ≤0.005, and ≤0.002, respectively (n≥3 independent experiments with ≥3 animals per group).

To analyze the effect of NKT cell stimulation on the course of infection, we injected 100 ng of αGalCer analog PBS57 at the time of infection. Interestingly, this led to a dramatic worsening of the course of disease, with lesion sizes reaching twice the size of the control group. Healing was also considerably delayed, prolonging the course of disease (Figure 3A). The effects correlated with parasite burdens over time, which were higher in the PBS57-treated group both in the ear (week 5) and spleen (week 8) (Figure 3B+C). Consistent with a primarily CD1d-mediated effect, PBS57 administration had only minor effects on the course of infection in CD1d^−/−^ mice (Figure 3D), where parasite burdens assessed in week 5 and 8 demonstrated only smaller changes (Figure 3E+F). Although not significant, antigen-specific cytokine levels showed a trend to a decrease of IL-4 production by CD1d^−/−^ LN cells than wild type counterparts and to an increase of IL-4 production in the C57BL/6 mice which had received αGalCer analog PBS57 (Figure 3G).

### Effect of αGalCer analog PBS57 is dependent on dose and time of administration

**Figure 4:**
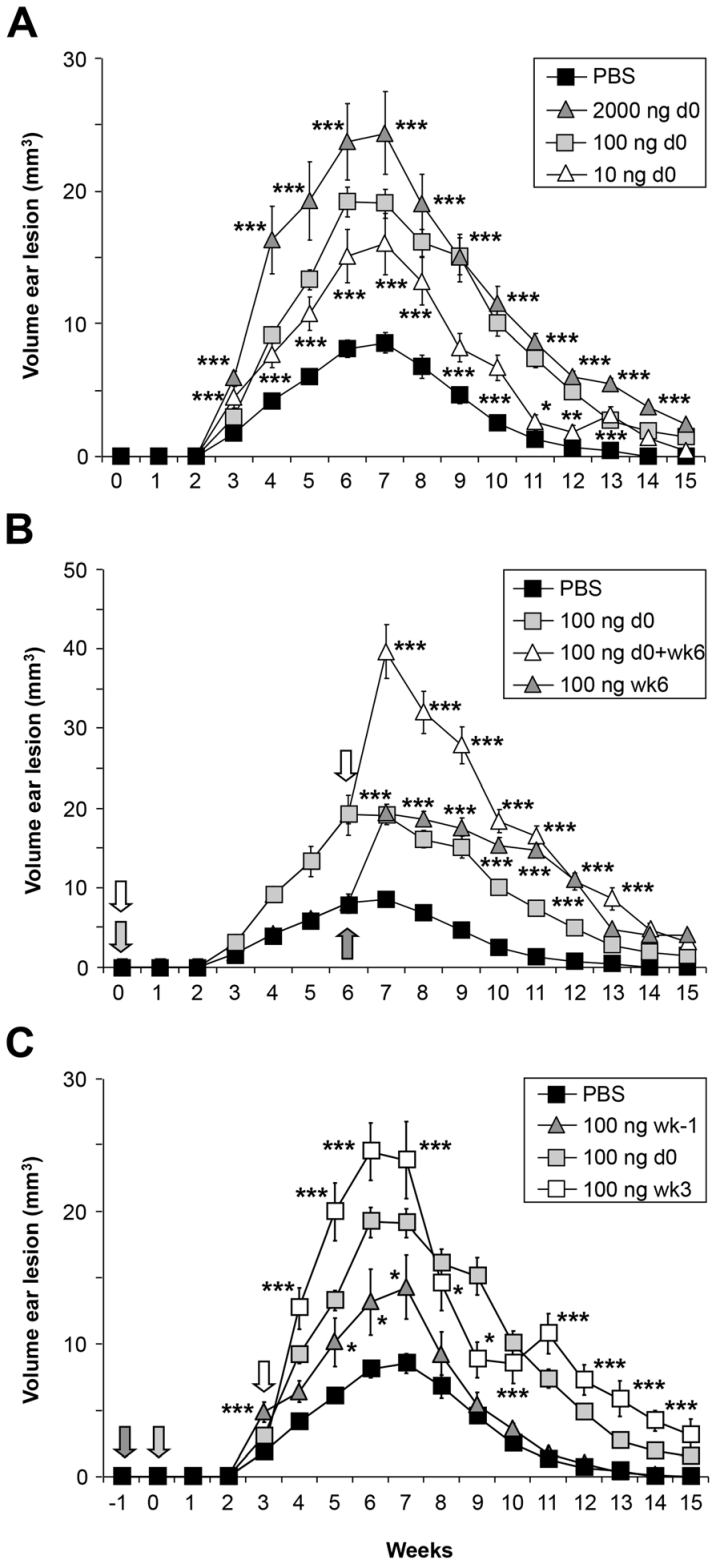
αGalCer analog PBS57 effects are dependent on dose and time of administration. **A.** Ear lesion volumes of wild type C57BL/6 mice infected with low dose promastigotes and receiving 10, 100, or 2000 ng. **B.** Ear lesion volumes during the course of infection with 100 ng of αGalCer analog PBS57 applied at the time of infection, 6 weeks after infection, or at both time points. **C.** As in B, only here PBS57 was applied at 1 week before and 3 weeks after infection. Significant differences between groups at each time point are labeled with *, **, and *** referring to p-values≤0.05, ≤0.005, and ≤0.002 respectively (n = 2 independent experiments with ≥4 mice/group).

Interested in assessing potential dose-dependent effects on the course of disease after intradermal *L. major* infection, αGalCer analog PBS57 was applied in doses of 10 ng, 100 ng, and 2 µg. Disease exacerbation upon PBS57 administration did show dose dependency with lesion size increases and prolongation of resolution being highest in the 2 µg-treated group and weakest in the 10 ng-treated group (Figure 4A). Administration of a subsequent dose of 100 µg at 6 weeks elicited a considerable effect with a rapid increase in lesion sizes and additional delay in healing. Finally, the control group treated with PBS57 only 6 weeks after infection showed a similar effect, considerably worsening the course of disease compared to the untreated group (Figure 4B).

To further delineate the time variable of PBS57 effects, an additional experiment with groups where 100 ng of PBS57 was applied 1 week before and 3 weeks after infection was initiated. Interestingly, while giving PBS57 one week before injection did slightly influence the course of infection, the effect was stronger in mice receiving PBS57 3 weeks after infection (Figure 4C). This confirmed the result of earlier experiments indicating that effects of αGalCer analog PBS57 were strongest if applied at the time of or post infection.

### αGalCer analog PBS57-induced effects are strain dependent and can improve disease in BALB/c mice

**Figure 5:**
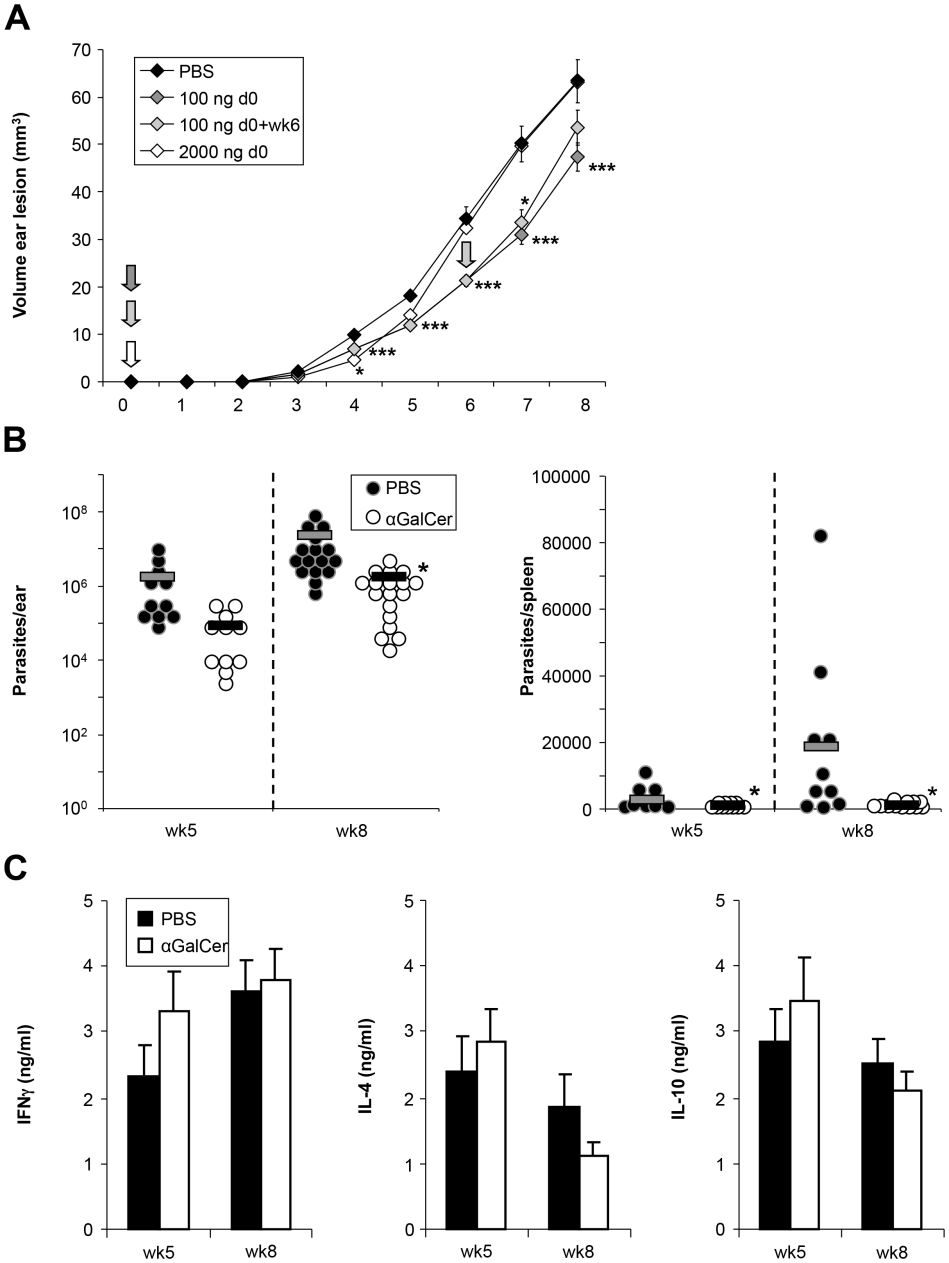
αGalCer analog PBS57 effects are strain dependent, improving disease in BALB/c. **A.** Ear lesion volumes of wild type BALB/c mice infected with low dose promastigotes and receiving 100 ng or 2000 ng at the time point of infection or 100 ng at the time point of infection and 6 weeks later. Significance is indicated as compared to control mice. **B.** Parasite burdens measured in the ear and spleen of control or 100 ng αGalCer analog PBS57 treated mice at week 5 and 8 post infection. **C.** Levels of IFNγ, IL-4 and IL-10 measured from lymphocytes restimulated with SLA, 5 and 8 weeks post infection. Results shown are from >1 experiment with a minimum of 4 animals per group. Significant differences between groups are labeled with *, **, and *** referring to p-values≤0.05, ≤0.005, and ≤0.002 respectively.

In contrast to resistant C57BL/6 mice, susceptible BALB/c mice routinely develop a Th2/Th17 response to *L. major* not allowing them to contain the parasite and ultimately leading to death due to extremely high visceral parasite loads. To analyze if αGalCer analog PBS57 administration in BALB/c mice has similar effects as in C57BL/6 mice, a range of concentrations were tested. Unexpectedly, the dose of 100 ng of PBS57 led to an improvement of disease. It did not allow the mice to contain infection, but significantly delayed the growth of lesions, resulting in prolonged survival (Figure 5A). This effect was not only seen in terms of lesion size, but also mirrored in parasite burdens, which were measured again at week 5 and 8 in ear and spleen (Figure 5B). IFNγ, IL-4, and IL-10 cytokine levels showed no significant differences (Figure 5C).

Application of a second dose of 100 ng of PBS57 led to a slight worsening of disease. In contrast, a single dose of 2 µg given at the time of infection showed only a small window of improvement from week 3–5 in terms of lesion sizes. At week 6, the lesion sizes were once again comparable to untreated controls (Figure 5A).

### αGalCer analog PBS57-induced cytokine secretion is strain-dependent

**Figure 6:**
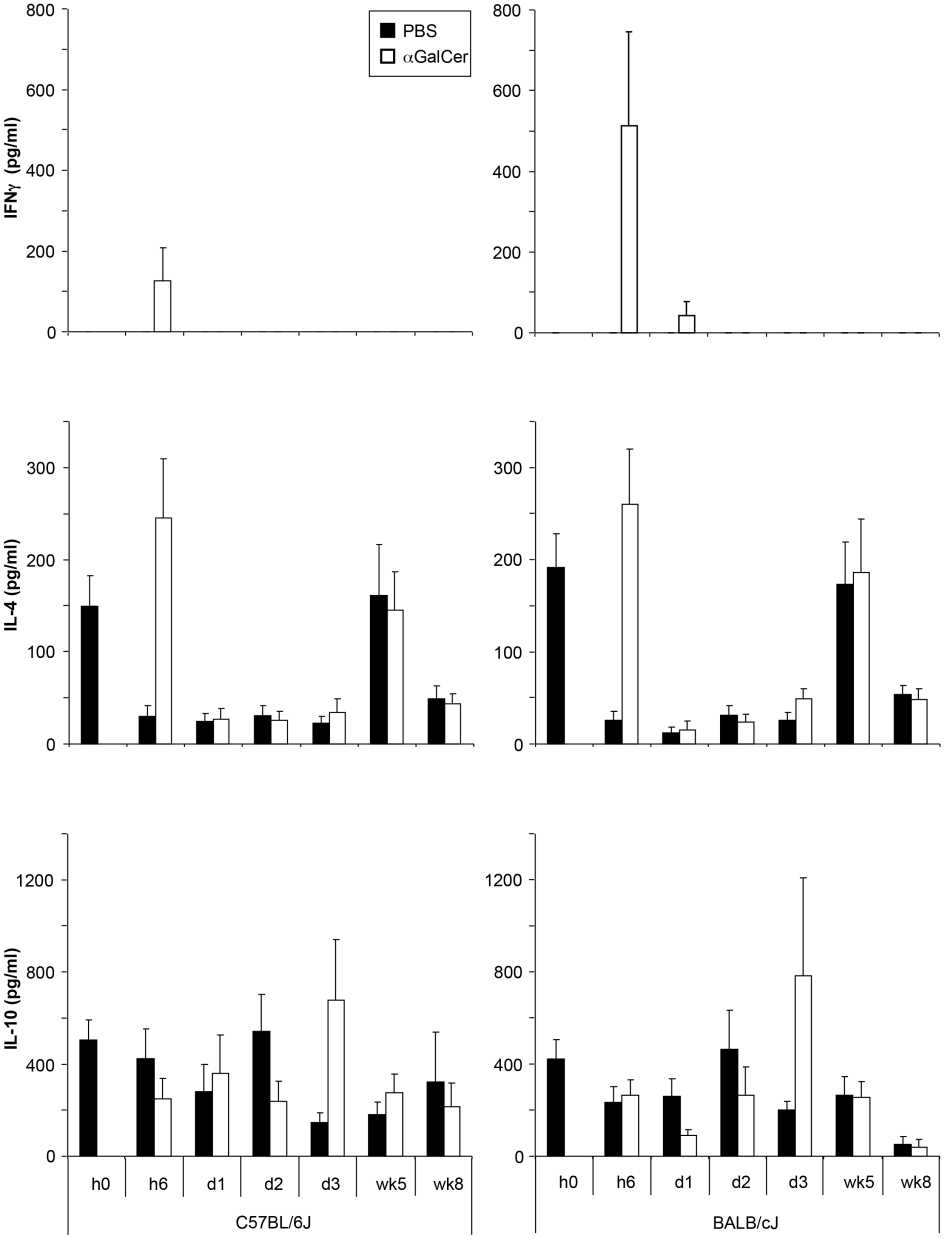
Cytokine secretion induced by αGalCer analog PBS57 is strain dependent. Serum levels of IFNγ, IL-4, and IL-10 were measured in the serum of C57BL/6 and BALB/c mice at different time points after infection. Mice received either PBS or 100 ng αGalCer analog PBS57 i.p. at the time point of infection with 10^3^ *L. major* promastigotes (n = 3 independent experiments with ≥8 mice per group). None of the differences noted were found to be statistically significant.

To determine if the differences seen in C57BL/6 and BALB/c infected mice were due to variations in the early cytokine response, we measured these in the serum shortly after *L. major* infection in PBS57 and untreated controls. PBS57 treatment led to an acute increase of serum levels of IFNγ and IL-4 at 6 hrs, which was not observed in untreated, infected controls. PBS57-treated mice also showed higher IL-10 levels 3 days post infection. The only clear observed strain-specific difference was a higher IFNγ level 6 hrs post infection in BALB/c than in C57BL/6 serum (Figure 6).

### Effects of αGalCer analog PBS57 in C57BL/6 mice are IL-4 dependent

**Figure 7:**
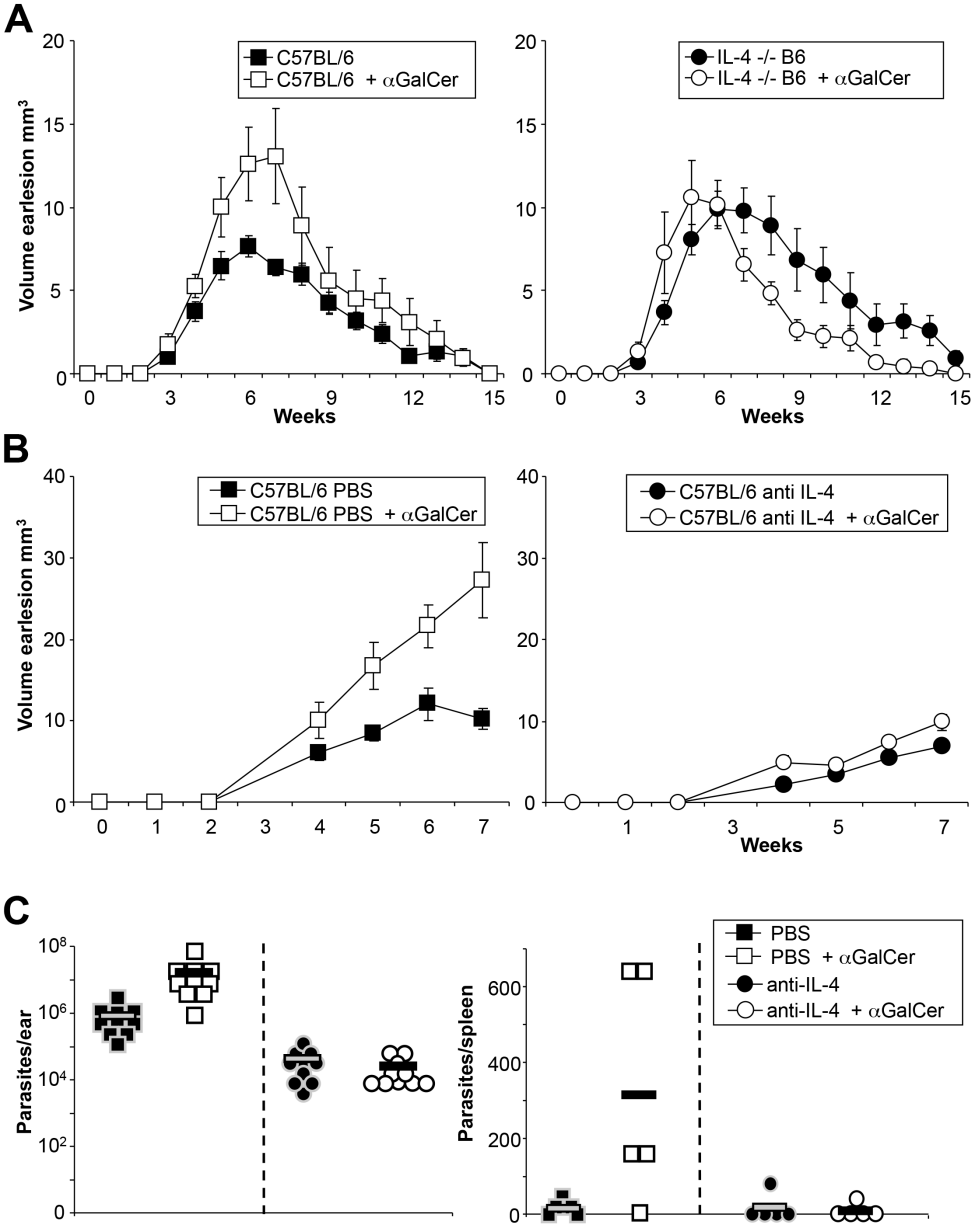
αGalCer analog PBS57 effects on C57BL/6 are IL-4 dependent. **A.** Ear lesion volumes of C57BL/6 mice infected with low dose promastigotes with or without 100 ng αGalCer analog PBS57 at the time point of infection. Wild type groups are shown on the left, IL-4-deficient mice on the right (n = 2 independent experiments, ≥4 mice per group). **B.** Ear lesion volumes of a similar experiment where mice received IL-4 neutralizing antibody (shown on the right, n = 1, ≥4 mice per group). **C.** Parasite burdens of mice from the experiment shown in B, 5 weeks post infection. Significant differences between groups are labeled with * referring to p-values≤0.05.

To further analyze the role of early cytokine secretion in the observed αGalCer analog PBS57-mediated effects on infection, experiments were performed eliminating IL-4. In one set of experiments, IL-4^−/−^ mice were infected with *L. major* and subsequently treated with PBS57. However, whereas controls showed the known exacerbation of disease after application of PBS57, this was not seen in IL-4^−/−^ mice (Figure 7A [23]). In another set of experiments, IL-4 neutralizing antibodies were applied to infected C57BL/6 mice. Here, similar results were observed, with PBS57 application no longer changing the course of infection (Figure 7B). Anti-IL-4-treated mice also exhibited significantly lower parasite loads. PBS57 administration no longer affected the course of infection in these mice (Figure 7C), supporting an important role of IL-4 in αGalCer analog PBS57-mediated disease exacerbation.

## Discussion

In the present study we demonstrated that NKT cells influence the course of *L. major* infection in a physiological low dose infection model. Mice on a C57BL/6 background lacking NKT cells showed an improved course of disease. Correspondingly, NKT cell stimulation with αGalCer analog PBS57 worsened the course of disease. Effects of stimulation were time, dose, and strain dependent. The effect of PBS57-stimulated NKT cells appeared to be IL-4 mediated, as neutralizing this cytokine abrogated the observed effects *in vivo*. Our results show that NKT cells should be considered both when treating active *Leishmania* infection as well as in the development of vaccines.

The reported effects of *L. major*-activated NKT cells observed in various models of *Leishmania* infection have been variable and often conflicting [10]–[16]. Most of this is probably due to both different infection models and *Leishmania* strains applied. In our model, 10^3^ infectious stage parasites are inoculated intradermally in the ear. This is considerably lower than in most other studies using *L. major*, which additionally often infect the foot pads instead of ear skin. Studies by Mattner et al. [13] and Ishikawa et al. [12] presented very different effects of NKT cells on the course of *L. major* infection than those we observed. In their hands, high doses of parasites (10^6^–10^8^) given intradermally or even intravenously showed a protective effect of NKT cells, with C57BL/6 mice having lower parasite loads than their NKT cell-deficient counterparts. We and others [24] argue that such high parasite doses are far removed from the actual real life scenario, where infection is initiated by small amounts of parasites leaving the sand fly during its feeding on the host. We therefore believe our findings might be more representative of most infection settings in humans.

Both NKT cell deficient mice strains on a C57BL/6 background controlled *L. major* infection significantly better than wild-type counterparts. CD1d^−/−^ mice lack all NKT cells. Jα18^−/−^ mice do not have the larger invariant Vα14-Jα18 TCR subset, however still maintain a smaller NKT cell subset which recognizes CD1d with a different TCRs. Although there was a trend toward better disease control for Jα18^−/−^ than CD1d^−/−^ mice (Figure 2) this was not statistically significant, nor were substantial differences in cytokine secretion between the two strains noted. Future studies would be needed to elucidate the role of different NKT cell subsets in *Leishmania* infection.

Stimulating NKT cells by applying αGalCer analog PBS57 significantly altered the course of disease. The effect was time- and dose-dependent. As initiating a Th1 immune response is critical for the control of *Leishmania* infection, we hypothesized that the mechanism through which NKT cells have a negative effect on the course of infection in C57BL/6 mice, could be through secretion of Th2 cytokines, in particular IL-4. When IL-4 was neutralized, either in IL-4 deficient mice or by applying IL-4 binding antibodies, the effect of PBS57 on the course of infection was alleviated. We believe this proves IL-4 secretion by NKT cells is the relevant factor negatively influencing control of *L. major* in C576BL/6 mice.

It is noteworthy that αGalCer analog PBS57 administration showed strain-dependent differences. While leading to disease exacerbation in C57BL/6, it improved the course of disease in BALB/c mice (at least in smaller doses of 100 ng). The higher amount of IFNγ secretion in the serum of PBS57-treated BALB/c we noted compared to C57BL/6 mice might play a relevant role. Additionally, we assume the effect could be strongly context-dependent, with the same cytokines (IFNγ and IL-4, both secreted by NKT cells), resulting in a slight Th1 shift in the developing Th2 response of BALB/c, compared to a Th2 shift in the Th1 setting of C57BL/6.

The strain specific observations are important to consider when trying to extrapulate what effect administration of αGalCer or its analogs might have in a human infection setting. Generally, it is believed that the course of infection seen in C57BL/6 mice better mirrors the situation seen in most humans with cutaneous leishmaniasis. As such, NKT cell activation through αGalCer analog PBS57 during the course of infection is most likely non-beneficial to the host and should be avoided. On the other hand, αGalCer has shown a very beneficial effect as an adjuvant in *L. major* vaccination studies [19]. This could mean that while applying αGalCer in a vaccination setting appears very promising, one should be careful before applying such a vaccine in the setting of an existing infection, as it could potentially lead to disease worsening.

A potential additional strategy would be to test other NKT cell stimulating glycolipids. PBS57, the compound used in our study, was shown to stimulate slightly higher amounts of IL-4 and IFN-γ secretion than the original αGalCer compound, KRN7000 [21]. Several versions of αGalCer and other glycolipids have been synthetically generated and vary in terms of cytokine response generated, favoring more of a Th-1 or Th-2 response [7], [25]. Potentially applying other αGalCer variants or glycolipid compounds which elicit a more pronounced Th1 profile cytokine stimulation could prove valuable, both in terms of treatment and vaccination for *Leishmania* infections.

In summary, our findings demonstrate that NKT cells influence the course of *L. major* infection in a physiological low dose model. The effects were strain-dependent and could be augmented through αGalCer analog PBS57 stimulation, leading to disease worsening in *Leishmania* resistant C57BL/6 mice. Our results make it apparent that immune response modulating effects of NKT cells should be considered both when treating active *Leishmania* infection as well as developing vaccines.

## Supporting Information

Table S1 Statistical analysis of data in Figure 1; frequency of CD4, CD8 and NKT cell populations in the course of infection.
(PDF)
