# Supplementary material for: Immune Modulating Effects of NKT Cells in a Physiologically Low Dose Leishmania major Infection Model after αGalCer Analog PBS57 Stimulation
Source: PLoS Negl Trop Dis. 2014 Jun 26;8(6):e2917. doi: 10.1371/journal.pntd.0002917 (PMC4072590; doi:10.1371/journal.pntd.0002917)
Supplement: Table S1 — Statistical analysis of data in Figure 1; frequency of CD4, CD8 and NKT cell populations in the course of infection. (PDF) [file pntd.0002917.s001.pdf]

Table S1: Statistical analysis of data in Figure 1; frequency of CD4, CD8 and NKT cell populations in the course of infection.

			CD4			CD8			NKT cells		
			wk3	wk5	wk8	wk3	wk5	wk8	wk3	wk5	wk8
C57BL/6	Ear	wk0	0,1674	0,1504	0,2529	0,1668	0,699	0,3089	0,0923	0,3528	0,0813
		wk3		0,1137	0,1656		0,2985	0,1872		0,4353	0,0258
		wk5			0,5713			0,1005			0,0397
	LN	wk0	0,0599	0,0015	0,0727	0,1416	0,0063	0,1187	0,0065	0,0267	0,103
		wk3		0,1437	0,2835		0,1851	0,7263		0,4302	0,2552
		wk5			0,0043			0,0513			0,3014
	Spleen	wk0	0,0015	0,0419	0,0662	0,0135	0,0006	0,0003	0,0238	0,174	0,9167
		wk3		0,0759	0,9001		0,0002	0,0002		0,5143	0,0268
		wk5			0,0434			0,882			0,1209
	Liver	wk0	0,0037	0,012	0,0009	0,0078	0,0002	0,0009	0,0017	0,1462	0,0004
		wk3		0,0062	0,0007		0,0096	0,5655		0,0307	<,0001
		wk5			0,0026			0,0029			0,0003
BALB/c	Ear	wk0	0,1473	0,1521	0,0174	0,3168	0,7815	0,0416	0,3399	0,5328	0,1493
		wk3		0,1618	0,0024		0,1539	0,0245		0,0668	0,088
		wk5			0,0058			0,0017			0,0162
	LN	wk0	0,1701	0,0099	0,0029	0,7419	0,774	0,4452	0,0681	0,2517	0,0084
		wk3		0,2955	0,2136		0,8921	0,731		0,9766	0,061
		wk5			0,8577			0,5597			0,0759
	Spleen	wk0	0,021	0,0007	0,0326	0,0608	0,0006	0,001	0,0407	0,1783	0,1423
		wk3		0,3739	0,19		0,0054	0,006		0,0228	0,0307
		wk5			0,3018			0,1098			0,4782
	Liver	wk0	0,2381	0,0034	0,0015	0,0667	0,0172	0,0173	0,0296	0,6627	0,1269
		wk3		0,0024	0,0011		0,1894	0,2089		0,0493	0,015
		wk5			0,9428			0,7227			0,0534

Shaded areas represent numbers where the p-value is <0.05
